# MAPseq: highly efficient k-mer search with confidence estimates, for rRNA sequence analysis

**DOI:** 10.1093/bioinformatics/btx517

**Published:** 2017-08-14

**Authors:** João F Matias Rodrigues, Thomas S B Schmidt, Janko Tackmann, Christian von Mering

**Affiliations:** Department of Molecular Life Sciences, and Swiss Institute of Bioinformatics, University of Zurich, Zurich, Switzerland

## Abstract

**Motivation:**

Ribosomal RNA profiling has become crucial to studying microbial communities, but meaningful taxonomic analysis and inter-comparison of such data are still hampered by technical limitations, between-study design variability and inconsistencies between taxonomies used.

**Results:**

Here we present MAPseq, a framework for reference-based rRNA sequence analysis that is up to 30% more accurate (*F*_½_ score) and up to one hundred times faster than existing solutions, providing in a single run multiple taxonomy classifications and hierarchical operational taxonomic unit mappings, for rRNA sequences in both amplicon and shotgun sequencing strategies, and for datasets of virtually any size.

**Availability and implementation:**

Source code and binaries are freely available at https://github.com/jfmrod/mapseq

**Supplementary information:**

Supplementary data are available at *Bioinformatics* online.

## 1 Introduction

Sequencing the DNA of microbial communities, either wholesale or after amplification of selected marker genes, has greatly advanced our understanding in many fields, including ecology, evolution and medical microbiology. However, the sheer amount of data and the conceptual and technical variability introduced by the wide variety of sequencing and analysis approaches pose difficult challenges for the consolidation and inter-comparability of findings, within and across studies.

The most widely used common denominator for inter-comparisons is taxonomy classification based on ribosomal RNA (rRNA), implemented in a number of software packages including RDP Classifier (Wang *et al.*, 2007), USEARCH (Edgar, 2010), VSEARCH (Rognes *et al.*, 2016) and NINJA-OPS (Al-Ghalith *et al.*, 2016), which are often bundled in broader pipelines such as MOTHUR (Schloss *et al.*, 2009) and QIIME (Caporaso *et al.*, 2010). However, these packages are either restricted to previously known taxa only, are suffering from computational limitations, or cannot be applied in a reference-mapping mode at the scales currently needed. Furthermore, approaches that are restricted to existing taxonomically classified reference sequences may not fully cover microbial diversity. This can be solved by including also unclassified reference sequences, pre-clustered into *operational taxonomic units* (OTUs)—and, ideally, these OTUs should be created at various different identity cutoffs and related to each other hierarchically. Such *hierarchical OTUs* (hOTUs) constitute an operational taxonomy in themselves and enable the assessment of taxa (even uncharacterized taxa) across different studies, at adjustable levels of granularity. MAPseq enables both, by providing a fast and accurate sequence read mapping against hierarchically clustered and annotated reference sequences. In addition to the software itself, we provide a large, curated reference of full-length rRNA genes, pre-clustered into hOTUs at different identity thresholds, and pre-classified to taxonomic categories based on the NCBI taxonomy and the All-species Living Tree Project dataset (Yilmaz *et al.*, 2014).

## 2 Results

**Fig. 1:**
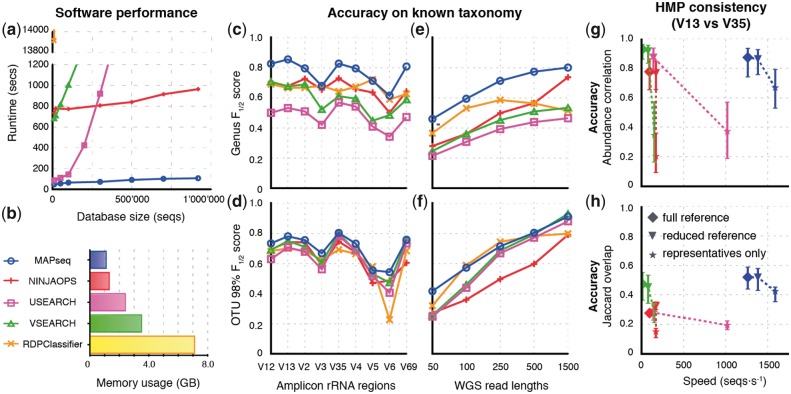
Benchmarking results on rRNA classification tasks. **(a)** Runtime complexity with increasing database size (using up to 8 CPU threads if supported). **(b)** Maximum memory usage during benchmarking. (**c–f)***F*_½_-scores for OTU (98%) or taxonomy (genus) classification, for fragments varying in length or rRNA region. Concordance tests on 2, 194 Human Microbiome Project samples, sequenced twice for both the V1V3 and V3V5 regions; **(g)** Pearson correlation of OTU abundances, and **(h)** overlaps in terms of OTUs identified

MAPseq is ten times faster than its closest competitor NINJA-OPS, and a hundred times faster than VSEARCH, on amplicon data (Fig. 1a ). Its memory requirements are lower than those of all other tools tested here (Fig. 1b). It can be used also on metagenomic shotgun sequence data, which it automatically searches for suitable rRNA sequences. Accuracy was benchmarked based on placements of reads of known identity, against hOTUs clustered at 98% identity (Fig. 1d and f) as well as against taxonomic categories at genus level (Fig. 1c and e). We also tested MAPseq‘s performance on reads of different lengths (Fig. 1e and f), as well as different hyper-variable regions of the rRNA gene (Fig. 1c and d).

With few exceptions, MAPseq outperformed all other tools, achieving a maximum *F*_½_-score (weighted harmonic mean of precision and recall) of 0.86 in genus mapping and 0.96 in OTU mapping. The accuracy increase over existing tools is most notable in genus classification, with 30% better *F*_½_-score at 500 bp long reads (Fig. 1e). For all methods, accuracy tends to increase with increasing read lengths, and hyper-variable regions within the rRNA gene yield better placement accuracy than reads originating within random positions within the gene. The increased computational efficiency and higher accuracy of MAPseq are due to several algorithmic innovations, including improved *k-mer* counting based on a pre-clustering step, full Needleman-Wunsch alignment of high scoring segment pairs, and a sensitive algorithm to compute classification confidence; see Supplementary Methods for details.

Strikingly, we find that even small reductions in the comprehensiveness of the reference dataset, by removal of near-identical sequences, can affect the accuracy for all methods tested (Fig. 1g and h;Supplementary Fig. S2). This shows that making reference datasets less redundant for runtime reasons has a significant trade-off cost in terms of mapping accuracy.

For an independent test of classification accuracy, we took advantage of a large data collection for which the very same samples had been subjected to two independent sequencing runs, using different regions of the rRNA gene (2194 samples from the Human Microbiome Project). Here, any good analysis framework should report strongly correlated results. As shown in Figure 1g, the correlations of abundances of mapped OTUs between sequencing runs were found to be fairly high for most methods; however, MAPseq achieved by far the best trade-off in terms of speed versus accuracy. We observed a similar trend in terms of the fraction of shared OTU identifications (Jaccard overlap, Fig. 1h): MAPseq resulted in the highest overlap (median = 0.52), followed by VSEARCH, NINJA-OPS, and USEARCH. Finally, we investigated the effect of using a different method for the *ab initio* clustering of reference sequences into OTUs. Using reference OTUs obtained with UCLUST (a widely adopted method) resulted in lower overall abundance correlations (median = 0.62) and Jaccard overlaps (median = 0.33) (Supplementary Fig. S3), independent of the software subsequently used for the mapping.

As a final validation, we have processed artificial ‘mock’ community data (Supplementary Fig. S4). We observe that MAPseq recovers their expected abundances better than other tools, at the species, genus and family levels.

In summary, MAPseq outperforms state-of-the-art methods dramatically in terms of speed, while also providing a more accurate and consistent approach. It can be used with the reference data provided, but also with custom references and/or taxonomies. MAPseq is open-source software implemented in multi-threaded C ++. Both the software and its reference data are available at: http://www.meringlab.org/software/mapseq/.

## Funding

This work was supported by the Swiss National Science Foundation (grant nr. 31003A-160095).

*Conflict of Interest*: none declared.

## Supplementary Material

Supplementary Data
